# Implementation of the Baby-Friendly Hospital Initiative in Mexico: a systematic literature review using the RE-AIM framework

**DOI:** 10.3389/fpubh.2023.1251981

**Published:** 2023-12-07

**Authors:** Angela K. Bueno, Mireya Vilar-Compte, Valeria Cruz-Villalba, Natalia Rovelo-Velázquez, Elizabeth C. Rhodes, Rafael Pérez-Escamilla

**Affiliations:** ^1^Department of Health, Universidad Iberoamericana, Mexico City, Mexico; ^2^Department of Public Health, Montclair State University, Montclair, NJ, United States; ^3^Research Center for Equitable Development EQUIDE, Universidad Iberoamericana, Mexico City, Mexico; ^4^Hubert Department of Global Health, Rollins School of Public Health, Emory University, Atlanta, GA, United States; ^5^Department of Social and Behavioral Sciences, Yale School of Public Health, New Haven, CT, United States

**Keywords:** breastfeeding, Ten Steps, BFHI, RE-AIM, Mexico, implementation research

## Abstract

The Baby-Friendly Hospital Initiative (BFHI) is a global strategy to encourage health facilities to promote, support, and protect breastfeeding by implementing a package of policies and practices known as the Ten Steps to Successful Breastfeeding. Prior studies have found that implementing the Ten Steps has a positive impact on breastfeeding outcomes. Yet, little is known about the implementation of the Ten Steps in Mexico. The objective of this study was to conduct a systematic review to evaluate the reach, efficacy/effectiveness, adoption, implementation, and maintenance of the Ten Steps in Mexico, using the RE-AIM framework. The systematic literature review included studies published in English or Spanish without date restrictions. Two of the authors coded each of the articles through a harmonized data extraction tool, and group meetings were used to discuss any discrepancies. The reviewed data were managed in the Rayyan platform. The risk of study bias was assessed through the Johanna Briggs Institute critical appraisal checklists. Of the 1,123 articles initially identified, 6 met the review inclusion criteria. None of the articles evaluated the reach and maintenance of the Ten Steps. The articles identified major gaps in the implementation of the Ten Steps. Most of the articles had important limitations in terms of their quality. In Mexico, it is necessary to rethink the BFHI and employ multiple strategies to improve implementation of the Ten Steps, including developing transparent BFHI monitoring mechanisms that produce data on implementation and that are publicly available, as well as investing in implementation research and evaluation to generate strong evidence to support the adoption and efficient maintenance of the Ten Steps in health facilities in Mexico. When properly implemented, BFHI becomes central to promote, protect, and support breastfeeding. Therefore, it is essential for Mexico to position BFHI as a top priority of the country's public policy agenda.

**Systematic Review Registration:**

identifier: CRD42021248118.

## 1 Introduction

The first 1000 days of life, from conception to the first 2 years, constitute a critical stage for healthy growth and development, in which breastfeeding (BF) plays a crucial role (1). Recent evidence has shown that suboptimal BF costs the world close to 1 billion dollars per day in lost productivity (2, 3). Similarly, according to the WHO, investing in promoting optimal BF practices, including initiation within the first hour of life, exclusive (EBF) for 6 months and continuing breastfeeding until the child is at least 2 years old, once nutrient-dense complementary foods get introduced at 6 months (2), could globally prevent the deaths of 820,000 children per year (2, 3).

BF is a personal maternal decision but that is bounded by multiple societal pressures and expectations that limit mothers' and caregivers' infant feeding decisions (4). From a socioecological perspective, such pressures and expectations are expressed through social, political, economic, organizational, and individual determinants (5–7). From this socioecological perspective, one of the many actors that influence mothers and caregivers' infant feeding decisions are health providers and the healthcare systems where they work. There is clear evidence that health providers need to be strongly engaged in BF protection, promotion, and support for BF programs to be effective (8). Given that health providers operate within healthcare systems, the standard operations procedures guiding the continuum of BF care in hospital and community environments, and the coordination between the two, are of the utmost importance for improving BF outcomes. This is because these standard operation procedures strongly influence the practice of health providers as well as breastfeeding decisions among mothers and their support networks (3, 9). At the end of the day, mothers need support and guidance in initiating, implementing, and maintaining optimal BF practices. If healthcare systems do not have skilled BF personnel and counseling programs, then mothers may not have access to the support they need and hence the agency to strengthen their BF self-efficacy, confidence, and motivation (9, 10). For this reason, the WHO and the United Nations Children's Fund (UNICEF) launched the Baby Friendly Hospital Initiative (BFHI) in 1989 based on the Ten Steps to Support Breastfeeding in Maternity Facilities and subsequent support and care at the community level (9, 11).

In many ways, the BFHI is a quality control system that allows maternity facilities to effectively support BF. Each facility that complies with the Ten Steps can eventually become accredited or certified as a “Baby Friendly Hospital” if they meet strict criteria including passing an external evaluation. Indeed, the BFHI provides an evidence-based accreditation program that promotes a series of steps aimed at: (i) planning BF during the prenatal stage, (ii) timely starting of BF in the perinatal period, and (iii) sustaining the exclusivity and duration of BF in the postnatal stage. To achieve this, the Ten Steps must be followed (Table 1) and should be aligned with trained health personnel and adequate hospital pre-, peri-, and postnatal practices. For the postnatal period, it is important to highlight that the tenth Step of the BFHI provides the extension to the Baby-Friendly Community Initiative (BFCI), which focuses on the community-based support needed after discharge. Despite its relevance, there is less evidence about the implementation of BFCI (12).

**Table 1:** Ten Steps to successful breastfeeding (BFHI), 1989 and 2018 versions.

**Step**	**Original version (1989)**	**Revised version (2018)**
1	Have a written breastfeeding policy that is routinely communicated to all healthcare staff.	(a) Fully comply with the International Code of Marketing of Breast-milk Substitutes and relevant World Health Assembly resolutions.
		(b) Have a written infant feeding policy that is routinely communicated to staff and parents.
		(c) Establish ongoing monitoring and data management systems.
2	Train all healthcare staff in the skills needed to implement the breastfeeding policy.	Ensure that all staff has sufficient knowledge, competencies, and skills to support breastfeeding.
3	Inform all pregnant women about the benefits and management of breastfeeding.	Discuss the importance and management of breastfeeding with pregnant women and their families.
4	Help mothers initiate breastfeeding within a half-hour of birth.	Facilitate immediate and uninterrupted skin-to-skin contact and support mothers to initiate breastfeeding as soon as possible after birth.
5	Show mothers how to breastfeed and how to maintain lactation even if they should be separated from their infants.	Support mothers to initiate and maintain breastfeeding as well as to manage common difficulties.
6	Give newborn infants no food or drink other than breastmilk, unless medically indicated.	Do not provide breastfed newborn infants any foods or fluids other than breastmilk, unless medically indicated.
7	Practice rooming-in, allowing mothers and infants to remain together 24 h a day.	Enable mothers and infants to remain together and to practice rooming in 24 h a day.
8	Encourage breastfeeding on demand.	Support mothers to recognize and respond to their infant's cues for feeding.
9	Give no artificial teats or pacifiers (also called dummies or soothers) to breastfeeding infants.	Counsel mothers on the use and risks of feeding bottles, teats, and pacifiers.
10	Foster the establishment of breastfeeding support groups and refer mothers to them on discharge from the hospital or clinic.	Coordinate discharge so that parents and their infants have timely access to ongoing support and care.

Sources: World Health Organization/United Nations Children's Fund Ten Steps to Successful Breastfeeding (original version: 1989 and revised version: 2018) (2).

In 2018, the BFHI steps, and especially the guidance on accreditation, underwent some adjustments to provide flexibility to countries on how best to implement the BFHI accreditation processes in their local contexts, but without sacrificing the reach and quality of implementation of each of the Ten Steps (13). Regarding the steps, an important modification was the need to specifically align maternity facilities with the WHO International Code of Marketing of Breastmilk Substitutes and the World Health Assembly-related resolutions (14). It also implicitly underlines the need to have well-documented standard operation procedures of the internal management information system to monitor the implementation of the Ten Steps in the facility.

The modifications made to BFHI in 2018 recognized the need for flexibility as the accreditation process works differently across countries. In some countries, such as the United States, it depends on a private institution (i.e., Baby-Friendly USA), but in other countries like Brazil and Mexico, the accreditation process is run by the government. The Ten Steps are evidence-based, as when properly implemented they have been shown to improve BF outcomes across the world region (15).

Nevertheless, implementation challenges still need to be better understood and addressed (16, 17). For example, even though the initiative is now over 25 years old, its coverage, measured as the proportion of children born in a BFHI-accredited hospital, remains very low (18, 19). In 2017, only 10% of newborns worldwide were delivered in BFHI-accredited hospitals (19). Previous studies have documented that countries have encountered difficulties in sustaining the BFHI because of financial and human resources considerations (14). It has also been noted that its successful implementation requires political commitment (12). Additionally, successfully implementing the Ten Steps can be challenging due to the lack of robust internal monitoring and evaluation systems at maternity facilities that can support quality assurance efforts related to the Ten Steps (14), including the training of health personnel (20, 21).

According to the experiences of some countries where the implementation of the BFHI has been relatively more successful, the BFHI requires adequate financing and flexibility to support its adoption, expansion, and maintenance at the national level (22). Consistently, these countries have identified the cost-effective training of health providers as being crucial for the success of BFHI rollout on a large scale (23), together with the internal monitoring and evaluation system mentioned above (9, 22).

This study aimed to conduct a systematic literature review of the BFHI in Mexico using the RE-AIM framework to organize the findings from the review (24). The RE-AIM is an implementation science framework that provides a structure for evaluating implementation (25). While all frameworks have limitations, they also provide the foundation for drawing from and developing a cumulative, evidence-informed science (26). In this sense, the RE-AIM allows to better understand how the BFHI has been adopted, implemented, and sustained, while considering its reach and effectiveness in improving breastfeeding outcomes. In fact, the RE-AIM has already been used to assess the BFHI in the United States and Brazil (17). Using the same framework to assess the same global initiative from an implementation science can lead to important cumulative lessons and may allow for comparisons to be made between studies (27). Hence, we expect that findings from this review can help inform Mexico and other countries about the major gaps in existing knowledge that need to be addressed to help guide the future implementation and scaling up of the BFHI at a national level in a way that is cost-effective and equitable.

### 1.1 The BFHI in the Mexican context

This systematic literature review focuses on the implementation of the BFHI in Mexico, as it is a good example of a country where the BFHI implementation has not gone according to plan. This in spite that in 1991 Mexico adopted the commitments of the World Summit for Children as part of the BFHI, and a national program called *Hospital Amigo del Niño y la Madre* (HANyM) was created, which incorporated the Ten Steps to improve BF indicators in the country (11). In 1993, maternity hospitals began to be certified at the national level through a government-run program. Between 1993 and 1999, 377 hospitals achieved the BFHI certification, but fewer than 42% (158) were recertified during that same period (28). Mexico faced several challenges with the implementation of the Initiative, including the lack of dissemination, monitoring, and maintenance plan. This led to a voltage drop; that is, the momentum was not maintained leading to a lack of coordination for the sustainability of a program. For example, during this period, Mexico experienced a deterioration in political will and support for BF promotion and protection, which was reflected in the lack of financing, intersectoral coordination, and relevant legislation to scale up and sustain the BFHI in the country over time (20, 29).

One of the objectives of the National Breastfeeding Strategy (ENLM, by its acronym in Spanish) 2014–2018 was to improve institutional competencies to support BF. The strategy proposed to increase the number of hospitals accredited as BFHI by at least 30% at a country level and obtain at least 180 Baby-Friendly Units at the first level of care (i.e., BFCI), but there is no public information to corroborate the achievement of these goals (30).

This deterioration process coincided with a period in which BF practices decreased in Mexico; between 2006 and 2012, there was a decrease in EBF from 22.3% to 14.4% at the national level (29). Due to multisectoral efforts put in place to address these declines in EBF, improvements in BF outcomes were reported by 2018–19, when EBF increased to 28.8% (31). Despite this improvement, Mexico is still far from the EBF goal established by the World Health Assembly for the year 2030 of 70% (32). The BFHI has not been systemically reactivated in Mexico, and considering the global evidence (22, 33), its reactivation is needed to continue improving BF outcomes in the country.

## 2 Methods

A systematic literature review (34) was carried out based on the Preferred Reporting Items for Systematic Reviews and Meta-Analyses (PRISMA) (35). The protocol was registered in PROSPERO before starting the search and analysis (N° CRD42021248118).

This review was guided by the RE-AIM framework, which includes five dimensions: (i) reach, which is defined as the number, proportion, and representativeness of individuals who are willing to participate in an intervention, (ii) efficacy or effectiveness of the intervention, (iii) adoption, which refers to the absolute number, proportion, and representativeness of settings and people who deliver the intervention who initiate an intervention, (iv) implementation, which focuses on fidelity to the intervention, its adaptations, and costs, and (v) maintenance, understood as the continuous implementation of the program at the setting level (i.e. sustainability of the Ten Steps) (24).

Guided by the RE-AIM, the review focused on two levels of results: implementation, and effectiveness and efficacy. Within the implementation results, we sought to identify the processes through which hospitals (or health subsystems) decide to adopt the Ten Steps, the barriers and facilitators to implementation, and the level of maintenance of the Initiative. In relation to effectiveness and efficacy, the review sought to identify the proportion of BFHI hospitals, the proportion of births that occurred in BFHI hospitals, and the differences in BF practices, skin-to-skin contact practices, knowledge about the Code, and BF training for health providers in BFHI vs. non-BFHI hospitals.

### 2.1 Search strategy

Systematic searches were carried out in four databases (Ovid MEDLINE, PubMed, Scopus, and Scielo), and in the UNICEF, WHO, Association of Certified Consultants in Breastfeeding (ACCLAM), and the National Institute of Public Health (INSP) websites. Additionally, a cross-referencing strategy was employed, which involved backward citation. To validate the search results, two local experts in the field were contacted via Zoom after the initial search to ensure no key documents had been omitted.

The search considered concepts related to the BFHI (baby friendly, BFHI), its Ten Steps (ten steps, 10 steps), and breastfeeding, in Spanish, English, and Portuguese. The complete search strategy can be found in Table 2. The country was not specified in the search, so as not to lose global studies that had data from the BFHI in Mexico.

**Table 2:** MeSH terms used in the systematic review.

**MeSH terms used in English**
“((Baby Friendly OR BFHI OR Ten Steps OR 10 Steps)) AND (Breast Fe OR Breastfe or Exp Breast Feeding))”
**MeSH terms used in Spanish**
“((Hospital Amigo OR IHAN OR Diez Pasos OR 10 Pasos)) AND (Lactancia OR Amamantar OR Extracción))”

### 2.2 Inclusion and exclusion criteria

Articles were included if they focused on one or more of the RE-AIM framework dimensions and if they were focused on public or private hospitals in Mexico with obstetric care that were either accredited or in the process of obtaining the accreditation of the BFHI (i.e., when they did not yet have the BFHI, but adopted it later) and were published in English, Spanish, or Portuguese up until February 2021. The review included quantitative or qualitative scientific papers and gray literature focusing on (i) people who had given birth to babies without medical conditions that could prevent initiation of BF, (ii) babies with information about their hospital of birth, or (iii) health professionals involved on the birth and perinatal services (see Table 3).

**Table 3:** Inclusion and exclusion criteria used in the systematic review.

**Inclusion criteria**
Studies were included if they focused on processes and impacts of the implementation of the BFHI in Mexico and if they met the following criteria:
(a) Any Mexican public or private hospital providing obstetric care, that was either in the process of obtaining the accreditation of the BFHI (that is, when they did not yet have the BFHI, but adopted it later) or were already BFHI-accredited
(b) Mothers who had given birth to babies without medical conditions that could prevent initiation of BF
(c) Babies with information about their hospital of birth
(d) Health professionals linked to neonatal hospital services
(e) Studies published in English, Spanish, or Portuguese up until February 2021
(f) Quantitative or qualitative indexed scientific papers and gray literature
**Exclusion criteria**
(a) Studies without information on the hospital's BFHI accreditation status (including whether they focused on mothers, births, or health providers)
(b) Studies focused solely or mostly centered on preterm infants, or on mothers with complications that limited initiation of BF
(c) Reviews and meta-analyses

Articles without information on hospital accreditation status (including whether they focused on mothers, births, or health providers) and studies focused solely or mostly centered on preterm infants or on mothers with complications that limited initiation of BF were excluded. Reviews and meta-analyses were also excluded.

### 2.3 Selection of articles and data extraction

Rayyan Systems (36) and Excel were used to perform the SLR. Studies and documents identified in databases and websites were initially imported into Excel to identify and remove duplicates. The remaining articles were then exported to Rayyan Systems (36). Three of the authors (AB, NR-V, and VC-V) screened the same first 20 articles and compared their screening decisions; if agreement was not reached or questions emerged, help from one of the senior authors (MV-C) was considered. Subsequently, they independently reviewed the titles and abstracts of articles to select which ones would be reviewed extensively (i.e., full text). Full texts were reviewed by two reviewers, and their inclusion in the SLR was determined by consensus.

#### 2.3.1 Quality evaluation

For the quality assessment, the checklists of the Joanna Briggs Institute (JBI) (34) were used because they have a wide variety of checklists according to the study designs, including one for cross-sectional studies.

#### 2.3.2 Data extraction

The results of the articles and documents selected for inclusion after full-text review were organized in a standardized data extraction table, which included the main characteristics of the documents (see Table 4), as well as information based on the dimensions of the RE-AIM (24) and the quality assessments as per the JBI quality assessment checklists (34).

**Table 4:** Data extraction guide.

**Data synthesis**
City/Subsystem (i.e., IMSS and ISSTE)
Sample size/population (i.e., medical doctors, nurses, and mothers)
BFHI steps assessed
Methodology (i.e., qualitative, quantitative, or mixed)
• Design
• Data collection mechanisms
• Aim/research question
• Type of analysis
Main findings
Quality assessment (JBI)

## 3 Results

### 3.1 Study characteristics

**Figure 1:**
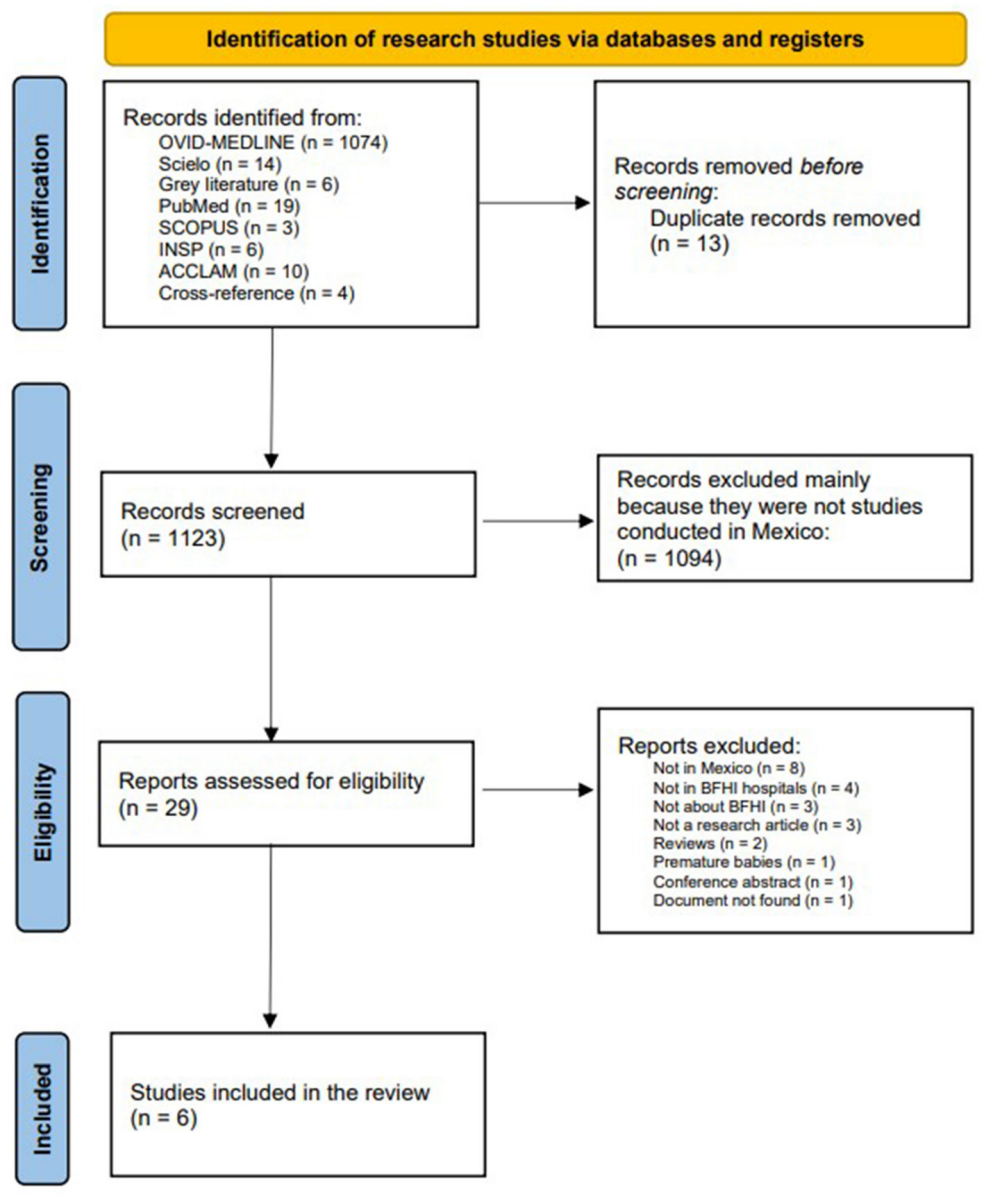
PRISMA for the identification of research studies.

Figure 1 summarizes the search results. Before starting the review, duplicate articles (*n* = 13) were eliminated, and then, the titles and abstracts were screened (*n* = 1,123), of which 1,094 were excluded, mainly because they presented findings from studies not conducted in Mexico. The authors reviewed the full text of 29 articles and eliminated 23. The reasons for exclusion were as follows: studies carried out in countries other than Mexico (*n* = 8), studies carried out in hospitals without the BFHI accreditation (*n* = 4), studies not related to BFHI (*n* = 3), non-scientific articles (*n* = 3), studies that were systematic reviews of topics related to BFHI (*n* = 2), studies carried out with a population of premature babies (*n* = 1), conference abstracts (*n* = 1), and studies not found (*n* = 1).

The six articles that were included in the review were divided into studies carried out in hospitals before obtaining the BFHI accreditation (pre-accreditation) (*n* = 3) and studies carried out in hospitals that already had the BFHI accreditation (post-accreditation) (*n* = 3).

#### 3.1.1 Pre-accreditation studies

Table 5 summarizes the information of the three pre-accreditation studies (37–39), all of which were conducted in hospitals in Mexico City. Two of the articles presented findings from studies that were carried out at the Luis Castelazo Hospital (37, 38), a tertiary care hospital for obstetrics and gynecology that belongs to the Mexican Institute of Social Security (IMSS). The third article focused on a study conducted at the Hospital General de México (39), which is a public tertiary referral hospital that offers obstetric care. These studies used different quasi-experimental study designs, including pre-post interventions (38, 39) and a cohort study with a comparison group (37).

**Table 5:** Studies conducted in hospitals before obtaining the BFHI accreditation (pre-accreditation).

**Author, year, city**	**Subsystem/ population**	**Design/main findings associated with BFHI steps**	**BFHI steps**	**RE-AIM components evaluated**	**Results related to BFHI steps**
Cisneros-Silva et al. (38), Mexico City	Luis Castelazo Ayala (IMSS) Obstetrics and Gynecology Hospital/Healthy binomials rooming-in, in a tertiary care hospital/250 without cesarean section and 250 with cesarean section (*n =* 500)	One-group pilot intervention/association between rooming in and initiation of BF	1, 2, 3, and 7	Adoption; Implementation	Rooming-in allowed ↑ consumption of human milk, 100% of newborns with rooming-in in the study were discharged after being breastfed. The children without rooming-in were discharged with a breast-milk substitute.
Vandale et al. (39), Mexico City	General Hospital of Mexico (HGM) / Training for pediatric and obstetric professionals (*n =* 110); BF sessions for primigravida women in the last trimester (*n =* 347); session on breast-feeding techniques for primiparous women + rooming-in (*n =* 423)	Quasi-experimental study with control group/initiation and duration of EBF	2, 3, 5, and 7	Efficacy/effectiveness; adoption; implementation	↑ knowledge in BF after training ( ≤ 12 h) (F+20.9267; *p < * 0.001 in ANOVA test); ↓ binomial separation time from 1.6 h to 1.3 h, ↑ number of children breastfed, 77.1% to 78.1%, ↑ number of times the child was breastfed, from 1.5 to 1.9 times; ↑ EBF from 52.4 to 54.9% and significant difference in age at full weaning, 12 weeks in control group and >17 weeks in intervention group.
Flores-Huerta and Cisneros-Silva (37), Mexico City	Luis Castelazo Ayala (IMSS) Obstetrics and Gynecology Hospital/Healthy binomials with term infants rooming-in (*n =* 29 born by cesarean section; *n =* 61 born by delivery) and without rooming-in (*n =* 31 born by cesarean section; *n =* 57 born by delivery) (*n =* 178)	Cohort/frequency of exclusive or partial breastfeeding	3, 5, 7, and 10	Efficacy/effectiveness; adoption	Regardless of the form of birth, rooming-in is the factor that influences the frequency of EBF the most. During the first month EBF was ↑ in the group rooming-in (61% vs 42%, *p < * 0.05); RR of EBF at 15 days: total rooming-in (1.62 [1.13–2.32]), births by delivery rooming-in (1.66 [1.04–2.66]); at 30 days: total rooming in (1.49 [1.09–2.04])

^1^(BFHI) Ten steps, Step 1. Fully comply with the International Code of Marketing of Breast-milk Substitutes; step 2. Ensure that all staff have sufficient knowledge, competencies, and skills to support breastfeeding; step 3. Discuss the importance and management of breastfeeding with pregnant women and their families; step 4. Facilitate immediate and uninterrupted skin-to-skin contact and support mothers to initiate breastfeeding as soon as possible after birth; step 5: Support mothers to initiate and maintain breastfeeding, as well as to manage common difficulties; step 6. Do not provide breastfed newborn infants any foods or fluids other than breastmilk, unless medically indicated; step 7. Enable mothers and infants to remain together and to practice rooming in 24 h a day; step 8. Support mothers to recognize and respond to their infant's cues for feeding; step 9. Counsel mothers on the use and risks of feeding bottles, teats, and pacifiers; step 10. Coordinate discharge so that parents and their infants have timely access to ongoing support and care. ^2^ BF Breastfeeding, ^3^ EBF Exclusive Breastfeeding, ^4^ RR Relative risk.

The pre-accreditation studies focused on research on steps 3 and 7 (37–39) of the BFHI, which focus on providing BF information and support to pregnant women and rooming-in post-delivery, respectively. Similarly, two of the articles addressed step 2 (38, 39), which focuses on ensuring that health personnel are trained to support and promote BF. Step 1, which is linked to the Code, was indirectly addressed in one of the articles (38) that reported findings from infant daily feeding records to register violations of the Code involving the use and promotion of breastmilk substitutes. Finally, one of the articles included step 10 (37), which focuses on postpartum follow-up of mothers and their children, that is, BFCI; specifically, this study addressed continuing care for mothers and their children on days 15, 30, 60, and 120 post-partum.

#### 3.1.2 Post-accreditation studies

The three post-accreditation studies (11, 40, 41) are summarized in Table 6. Two of them were carried out in Mexico City (11, 40), one at the Hospital General de Zona 1 “A,” belonging to the IMSS (41), and the other one at the Hospital General de Mexico (11). The third study was conducted at the IMSS Hospital General de Zona IV No. 8 (41) in Ensenada, Baja California.

**Table 6:** Studies conducted in hospitals after obtaining the BFHI accreditation (post-accreditation).

**Author, year, city**	**Subsystem/ population**	**Design/main findings associated with BFHI steps**	**BFHI steps assessed**	**RE-AIM components evaluated**	**Results related to BFHI steps**
Thompson-Chagoyán et al. (40), Mexico City	IMSS Area 1 “A” General Hospital/Review of reports of consumption of breastmilk substitutes, months before the start of the BFHI program (period A), and months after (period B) (*n =* 22 months)	Cross-sectional/consumption of breastmilk substitutes	6	Efficacy/effectiveness	Significant differences (*p < * 0.001); reduction in the number of containers, kilograms, costs, and liters of breastmilk substitutes offered, as well as in costs per child in period B
Navarro-Estrella et al. (41), Ensenada, Baja California	IMSS Area IV General Hospital No. 8/healthy working mothers, beneficiaries of this hospital, with healthy single babies with gestational age ≥37 weeks (*n =* 265)	Cross-sectional/early abandonment of BF	3 and 10	Efficacy/effectiveness	Group I: mothers with early abandonment in BF; group II: mothers who prolonged BF for more than 3 months. 42.3% (*n =* 112) of the mothers abandoned BF early; the risk factors for early abandonment were: wrong knowledge of BF (OR 5.97, CI 1.67–20.67); not having breastfed before (OR 2.98, CI 1.66–5.36); previous BF planning for only 0–3 months (OR 16.24, CI 5.37–49.12); lack of facilities in the work environment (OR 1.99, CI 1.12–3.56)
Hernández-Garduño and Rosa-Ruiz (11), Ciudad de México	General Hospital of Mexico (HGM)/educational intervention on BF, with initial and final evaluations, in nursing staff; attendance to the course was by direct indication or personal interest (*n =* 152)	Pre- vs. post-evaluation, one-group pilot intervention/changes in knowledge on BF	2	Implementation	Significant results comparing the knowledge evaluations before and after the training on BF (*p < * 0.001) in all levels of professional training. The training lasted 18 h, including 6 h of clinical practice; the thematic content was supported by educational material on BF developed by the Ministry of Health and UNICEF.

^1^ (BFHI) Ten steps, step 1. Fully comply with the International Code of Marketing of Breast-milk Substitutes; step 2. Ensure that all staff have sufficient knowledge, competencies, and skills to support breastfeeding; step 3. Discuss the importance and management of breastfeeding with pregnant women and their families; step 4. Facilitate immediate and uninterrupted skin-to-skin contact, and support mothers to initiate breastfeeding as soon as possible after birth; step 5. Support mothers to initiate and maintain breastfeeding as well as to manage common difficulties; step 6. Do not provide breastfed newborn infants any foods or fluids other than breastmilk, unless medically indicated; step 7. Enable mothers and infants to remain together and to practice rooming-in 24 h a day; step 8. Support mothers to recognize and respond to their infant's cues for feeding; step 9. Counsel mothers on the use and risks of feeding bottles, teats, and pacifiers; step 10. Coordinate discharge so that parents and their infants have timely access to ongoing support and care. ^2^ BF, breastfeeding; ^3^ OR, odds ratio; ^4^ CI, confidence Interval.

No pattern was observed in the Ten Steps that were addressed. The study carried out at the Hospital General de Zona I “A” IMSS evaluated step 6 (40), which is related to not providing any food or liquid to breastfed newborns, unless it is medically indicated. On the other hand, the study carried out at the Hospital General in Ensenada considered steps 3 and 10 (41), linked to providing information on good management of BF to pregnant women and to postpartum follow-up for mothers and their children. The study carried out at the Hospital General de Mexico analyzed step 2 (11), which emphasizes the importance of health personnel knowledge and skills to support BF.

#### 3.1.3 Pre- and post-accreditation studies from the perspective of the RE-AIM

**Figure 2:**
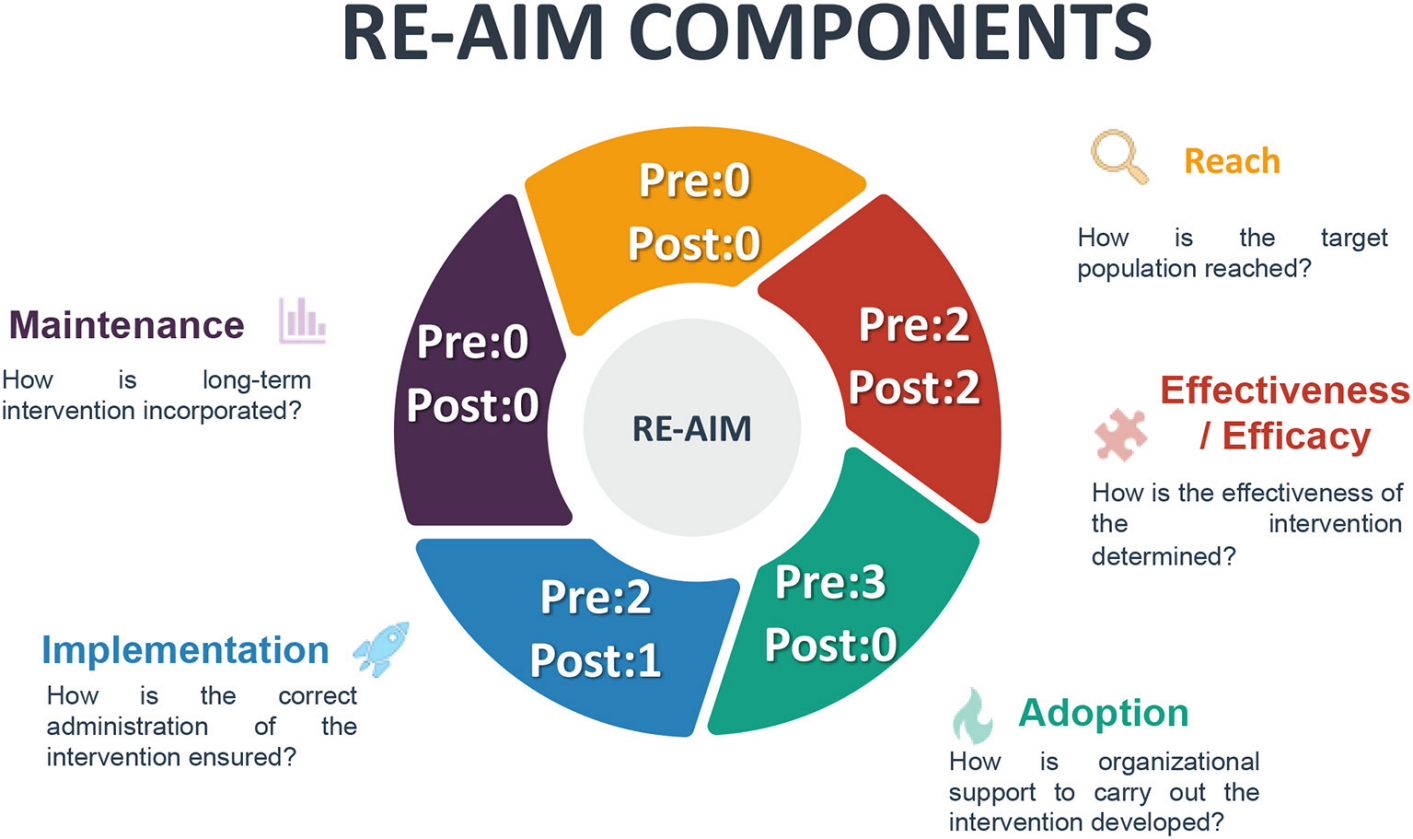
Frequency of the RE-AIM model components in the pre–post-accreditation studies.

Figure 2 shows the distribution of studies according to the RE-AIM analyzed dimensions (24) and underlines the lack of literature around the reach dimension. As such, no assessment of the proportion of accredited hospitals nor the proportion of births occurring in these hospitals have been published in the peer-reviewed literature.

Studies focused on the efficacy (*n* = 4) of the BFHI in Mexico reported positive impacts including a reduction in the average time of separation of the mother–child from 1.6 h to 1.3 h (33), higher frequency of EBF due to rooming-in (37), and a reduction of hospital costs linked to purchasing breastmilk substitutes (40).

Adoption of the Ten Steps was only analyzed in pre-accredited hospitals (*n* = 3). These articles focused on documenting new practices, such as rooming in and giving information to pregnant women about the importance of BF (37, 39). However, they did not delve into the management aspects that facilitated or led to the adoption of the Ten Steps.

Among the articles that documented the implementation of the Ten Steps (*n* = 3) (11, 38, 39), it was emphasized that such Steps helped in improving hospital routines and in identifying areas for improvement. For example, the Hospital Luis Castelazo worked to establish rooming-in, even though it is a high-risk hospital (37, 38). It began by training gynecology and obstetrics health personnel to increase decision-making skills with greater precision regarding when rooming-in should be indicated, continued, or suspended. In addition, the Hospital implemented a program to motivate the staff to acknowledge the importance of BF for both the mother and the child's health (38). The Hospital General de Mexico also provided training to the nursing staff of different shifts and services, either by indication of their immediate superior or out of personal interest. It established courses of a total duration of 18 h with 6 h of supervised clinical practice and followed guidelines established by the Ministry of Health and UNICEF (11).

### 3.2 Evaluation of the quality of the studies

This SLR identified that the quality of the evidence could be improved. Two cross-sectional studies were analyzed. One study evaluated the effect of a program on the consumption of breastmilk substitutes at a hospital (34). According to the JBI checklist (34), this study did not meet any of the established criteria, for which it was determined as a very low-quality study. There was no clear description of the inclusion and exclusion criteria of the sample, no confounding factors were identified, and the data collection process was not explained, which affects the validity and reliability of the study. On the other hand, another study at the Hospital of Ensenada, Baja California (41) was considered of acceptable quality despite the risk of incurring recall bias by applying a retrospective questionnaire during the postpartum stage about which were the mothers' feeding plans while pregnant.

Three studies were quasi-experimental. One evaluated BF training for nursing staff (11), another one referred to rooming-in and BF initiation in a tertiary care hospital (38), and another one assessed a BF promotion program at the HGM (39). These were considered to be of low quality. None had a control group, limiting the validity of causal inferences.

Finally, a cohort study evaluating rooming-in and EBF (37) showed confusing criteria. Data such as exposure measurements, allocation of exposed and unexposed groups, and confounding factors were not specified. Additionally, the study incurred in loss to follow-up of ~20%, compromising the internal and external validity of the study. For these reasons, this study was deemed to be of low quality.

## 4 Discussion

This systematic literature review, based on the RE-AIM framework, provided a structured approach toward understanding BFHI gaps in Mexico. Through its orientation in process results and impact, it showed which barriers and facilitators were contributing to the progress of the implementation of Ten Steps in Mexico as well as the knowledge gaps with respect to the Initiative. Ultimately, it showed the need to have consistent methods to investigate, evaluate, and follow up on BF and BFHI indicators that allow for maximizing the benefits of the Initiative in the country.

Globally, there is sufficient evidence of the positive impact of the Ten Steps on BF outcomes, including the tenth step, which refers to the community-level follow-up and support, i.e., BFCI (15). However, it has also been highlighted that implementing such Steps can be challenging and implementation science can contribute to making sense of when, where, and why the Ten Steps are being implemented or not, and to help better realizing the impact of such evidence-based intervention. Given that in Mexico there have been challenges with hospitals sustaining the Ten Steps over time, this SLR sought to document the existing scientific evidence around the implementation of the BFHI and its Ten Steps. A substantial lack of evidence was found. Only six studies were identified, which reveals there is very little information about the BFHI in Mexico. Moreover, the quality of the published studies was, on average, low. Regarding the tenth step, while the BFCI has been recognized as a relevant practice by the Mexican Ministry of Health, there is a profound lack of evidence about its adoption and implementation. While in Mexico most deliveries happen within a medical context in which the BFHI is fundamental (42), the postpartum follow-up takes place at the primary level and the community in which the adequate implementation of the BFCI is crucial. The community approach needs to be embraced as infant feeding decisions depend on multiple determinants and actors (43, 44).

In Mexico, there is no information indicating the processes by which hospitals or health subsystems decide to adopt or implement the BFHI and BFCI. These are relevant data to make contextual adaptations, scale up good practices, follow up to monitor progress, and identify strategies to improve implementation of the Ten Steps. In the current review, no study in Mexico with a focus on long-term results was found. Therefore, the continuity of the Initiative and its review and control processes are unknown. There are scarce published data regarding the number of accredited hospitals. While some rates are cited in prior reports (28), no official source specifying the status of the implementation of the Ten Steps was found, precluding the establishment of areas of opportunity to strengthen the program.

Mexico could benefit from practices implemented in other countries. For example, in Brazil, the Ministry of Health established a monitoring tool that allows access to information such as data, evaluations, and results of all hospitals. This monitoring tool allows for evaluating what is being implemented. In addition, hospitals that have the BFHI accreditation operate a self-management process carried out by their own health personnel (17, 22). In the United States, the BFHI is supervised by Baby-Friendly USA, an independent accreditation body that monitors the number of babies born in hospitals that have adopted the Ten Steps. In addition, the US Centers for Disease Control and Prevention (CDC) has provided financial support to health departments to increase the adoption of the Ten Steps in hospitals across the country (21, 45). They also conducted a survey on maternity, nutrition, and childcare practices (mPINC) (45), and a national census of maternity practices in order to identify areas of opportunity to improve the implementation of the Ten Steps and increase BF rates (45). The experience of countries like Kenya in the implementation of the BFCI can also help in understanding the relevance and implementation strategy to care for mothers and their infants after birth, from the health facility to the community where community health volunteers are fundamental to support and improve breastfeeding (46).

During the last 20 years, the ENSANUT has documented the national rates of BF in Mexico. A critical next step is to close information gaps around the implementation of the Ten Steps (47), including the compliance with the Code of Marketing of Breastmilk Substitutes, which could not be really assessed as all the studies were prior to the 2018 modification of the Ten Steps. The Becoming Breastfeeding Friendly Index Committee in Mexico (BBF-Mexico) (47, 48) has tried to obtain information about the number of births that occur in accredited hospitals, but there is no data on how many children have benefited from the Initiative, limiting the assessment of the reach of the program. BBF-Mexico has further underscored the absence of public data on the number of accredited hospitals, which makes it difficult to assess the maintenance of the Initiative (47) and coincides with the SLR findings from a RE-AIM perspective.

According to the BF gear model (BFMG) (20, 49), a model that identifies eight “gears” (i.e., legislation, advocacy, research, funding, promotion, training, political will, and coordination) that must work in harmony for effective support and promotion of BF, Mexico has some important gaps. Recently, the BBF-Mexico Committee warned that several of these gears are not working correctly (44, 49), including hospital practices and BF training for the health workforce (50). Previous studies in Mexico have also found that knowledge of the Code of Marketing of Breastmilk Substitutes among health professionals is severely lacking (18, 51). This is worrisome as large violations of the Code have been documented in Mexico, and health professionals have been found to play a role in these violations (51–53). BFHI steps 1 and 2 represent an opportunity to address these issues and therefore help women be better informed about BF through extensively trained staff.

Because there is no publicly available data on the BFHI in Mexico, transparency regarding the implementation of the Ten Steps is extremely limited. The implementation of the BFHI depends on its nomination granted by IMSS or the Health Ministry (SSA), which is similar to the Brazilian model (22); however, the designation and re-evaluation system is not public and, thus, difficult to follow. Based on data obtained by formal request in 2019 to the Ministry of Health, < 11% of maternity hospitals at the national level had been certified in the previous 5 years. There were only 121 baby-friendly hospitals nationwide, of which 85 were accredited at the time of data collection (49).

It is known that the BFHI represents more work for health personnel, who are often already overextended. Therefore, it is necessary to generate incentives to encourage accreditation, maintain it, and rethink the accreditation mechanism (54). For example, the health system of Vietnam established Hospital Quality Assessment Criteria (54), which works by establishing points at the national level that seek to improve the quality satisfaction and safety of patients. Criteria include BF communication, training, and practices. This model implies the strengthening of internal monitoring systems that are targeted at helping hospitals and their staff improve internal management, processes, and practices.

While a potential limitation of this systematic review is its narrow geographic focus, it also contributes to the broader literature on the implementation of the BFHI and BFCI through the RE-AIM framework, which has previously been used in Brazil and the United States. The implementation lens will allow us to document what and how has worked (or not) in scaling and sustaining the Ten Steps.

## 5 Conclusion

In Mexico, it is necessary to rethink the BFHI. It is fundamental to generate public follow-up and monitoring mechanisms to better understand what the adoption and implementation challenges are. Equally, it is necessary to propose management models that promote the adoption and sustainability of the Ten Steps considering the challenges of the national health system. In Mexico, the BFHI and the BFCI can be key factors in the promotion, protection, and support of BF, but it is necessary to bring the issue forward to the public policy agenda to identify the reasons why the Initiative has not worked and look for effective strategies to improve its implementation, monitoring, and evaluation.

## Data availability statement

Since this article is a systematic review, data comes from articles in academic journals that have been published in the public domain. Data sharing is not applicable to this article.

## Author contributions

AB conceptualized the systematic review, reviewed abstracts, titles, and manuscripts, participated in synthesis tables, and drafted the full manuscript. MV-C conceptualized the systematic review, developed and tested the search strategy, provided guidance in dissenting and inclusion about specific studies and drafted the full manuscript, and participated in synthesis tables. VC-V drafted the protocol for the systematic review, conducted the search, reviewed abstracts, titles, and manuscripts, and participated in synthesis tables. NR-V conducted the search, reviewed abstracts, titles, and manuscripts, and participated in synthesis tables. ER and RP-E provided a critical review of the review protocol and the full manuscript.
